# Prevention and Epidemiological, Clinical, and Economic Issues of COVID-19: Far More Than a Respiratory Disease

**DOI:** 10.3390/jcm11237218

**Published:** 2022-12-05

**Authors:** Giuseppe La Torre, Luca Moretti, Francesca Vezza

**Affiliations:** Department of Public Health and Infectious Diseases, Sapienza Universitya of Rome, Piazzale Aldo Moro 5, 00185 Rome, Italy

The *Journal of Clinical Medicine* has published many papers on coronavirus disease 2019 (COVID-19), and it is now clear that this is not simply a respiratory disease. This editorial focuses on the clinical, epidemiological, and economic issues related to COVID-19, as well as its prevention.

COVID-19 is a systemic disease characterized by a disproportionate inflammatory response in the acute phase [1]. SARS-CoV-2 primarily affects the respiratory system and its severity ranges from asymptomatic infection to severe acute respiratory distress. Neurological symptoms, including anosmia and ageusia, are frequent, and some patients may also present with cutaneous and gastrointestinal symptoms [2]. Anosmia and ageusia are predictive symptoms of COVID-19 and are useful indicators for its early detection, as are fever, cough, and dyspnea. In particular, these symptoms have high specificity and negative predictive values [3]. Moreover, thrombotic and thromboembolic diseases appear to be frequent complications in COVID-19 patients. Consequently, the severity of the disease may vary depending on the clinical presentation and the organs affected. In addition, the severity and mortality rate are related to many host factors, including age, gender, chronic conditions, comorbidities, race, and ethnicity [2]. Females are more susceptible to post-COVID symptoms [4]. Long COVID is a complex syndrome with protracted heterogeneous symptoms, and the WHO has established the following clinical case definition of post COVID-19 syndrome: “it occurs in individuals with a history of probable or confirmed SARS-CoV-2 infection, usually 3 months from the onset of COVID-19 with symptoms and that last for at least 2 months and cannot be explained by an alternative diagnosis” [5]. Post-COVID symptoms are present in hospitalized COVID-19 survivors months after infection. Females are at a higher risk for developing long-term post-COVID symptoms including anxiety, fatigue, dyspnea, depression, and reduced sleep quality than males. Female sex is also a risk factor for the development of some long-term post-COVID symptoms, including mood disorders. Other risk factors suggested in the literature to be associated with post-COVID symptoms include age, a greater number of COVID-19 symptoms in the acute phase, a longer hospital stay, and more medical comorbidities [4]. Many COVID-19 survivors continue to experience respiratory symptoms, and several studies have reported abnormalities in pulmonary function tests and chest CT images, even months after hospital admission [5]. The CPET (Cardio-Pulmonary Exercise Test) data in symptomatic patients are compatible with ventilatory inefficiency (high VE/V_CO2_ with a lower PET_CO2_), which is strongly suggestive of a ventilation/perfusion mismatch due to pulmonary vasculopathy and is also supported by a mild reduction in DL_CO_ [1]_._ Dyspnea and cough are the most frequently described respiratory symptoms. The most common abnormality is diffusion impairment, followed by restrictive ventilatory defects. One of the possible complications of ARDS is pulmonary fibrosis. The risk of developing this condition is related to the cellular mechanisms that occur in response to acute lung injury, and it can lead to an abnormal and persistent inflammatory response and excessive proliferation of fibroblasts. Persistent endotheliopathy, which results in a pro-coagulant state and inflammatory cytokine production, could be involved [5]. Intravascular fibrin aggregates in pulmonary vessels are the most common finding, regardless of the type of pulmonary injury; endothelial dysfunction has previously been linked to activation of the coagulation cascade in COVID-19 patients. Moreover, intussusceptive angiogenesis has been also observed in the lungs of COVID-19 patients, which may disrupt the microcirculatory structure and has been previously described in chronic thromboembolic pulmonary hypertension [1]. Finally, despite the severity of the initial infection, persistent fatigue seems to be the most bothersome symptom of post-COVID-19 syndrome. During acute SARS-CoV-2 infection, pain as a symptom usually manifests as headache, sore throat, or arthralgias/myalgias. Pain problems are found mostly in the head/face, chest, spine, and extremities or as migrating pain. Musculoskeletal pain and headache (probably of neuropathic origin) are reported to dominate, although even visceral pain (IBS-like symptoms and irritable urinary bladder) could be indicative of the condition. Different types of pain might be seen in the same patient suffering from post-COVID-19 syndrome. In one study, although the pain intensity was moderate, many participants who were healthy before infection developed pain, which might be considered a risk factor for chronic pain. Those with comorbidities before COVID-19 infection consumed more medications after infection and tended to develop widespread pain/fibromyalgia. The total number of medications might simply reflect an increased range of comorbidities following COVID-19 infection [6].

Patients infected with SARS-CoV-2 appear to have a higher incidence of thrombotic events. It was found that SARS-CoV-2 infection could cause AIS among a considerable number of young and mostly male patients who did not have vascular risk factors. The majority of these young patients had embolic-appearing stroke on their neuroimaging. Stroke in older patients can be attributed to existing vascular risk factors, which is no different to the population studies conducted prior to the SARS-CoV-2 pandemic [7].

During the COVID-19 pandemic, compared to non-COVID patients, there was a higher hospitalization rate and a longer average hospital stay in COVID-19 patients; together with the extensive use of cortisone and antibiotics for COVID treatment, this represents a risk factor for nosocomial infections, including Clostridium difficile infections (HO-CDI). Despite this, the HO-CDI rate among COVID-19 patients was within the range of incidence observed in reports performed before the COVID-19 pandemic. The disadvantage is that CDI worsens the outcome of COVID. 

CDI COVID-19 patients, in comparison with those without CDI, have greater severity of COVID-related symptoms, a longer mean length of in-hospital stays, higher mortality, and more complications at discharge [8].

COVID-19, especially in the first wave, was a disease characterized by high mortality. Italy was the first country in Europe to be affected by coronavirus 2019. As of 28 May 2020, a total of 31,851 deaths in SARS-CoV-2—positive patients were reported in Italy. Age showed a correlation with mortality, with most deaths occurring in the elderly population: 55.8% were over 80 years old and 36.5% were between 60 and 79 years old, while only 5.7% were aged 39–59, and there were no deaths under the age of 30. Comorbidities were observed in a high percentage (72%) of deaths in people who tested positive for SARS-CoV-2, suggesting that death was often the result of the concomitance and interaction of different chronic diseases with SARS-CoV-2 infection. Nevertheless, COVID-19 was the cause directly leading to death in the vast majority (88%) of cases. Moreover, in a considerable proportion of deaths (28%), and also in younger people, no comorbidities were reported, suggesting that this condition can be fatal in healthy people [9].

The COVID-19 pandemic required health systems to adapt to new care needs. Most hospitals adopted extraordinary measures, including resource reallocation, ward repurposing, and work reorganization. These interventions caused frequent delays and interruptions to ordinary care, particularly for people affected by non-communicable diseases (NCDs) [10].

Many countries have taken extraordinary measures to contain the COVID-19 pandemic, such as implementing lockdown, which negatively impacted people’s emotional functioning and increased levels of stress and symptoms associated with anxiety and depression. The 8 weeks of lockdown in Spain initially had a negative impact on people’s psychological health. However, symptoms of depression and anxiety improved throughout the weeks. Meanwhile, drug use, satisfaction with life and health, and optimism about personal and societal futures worsened. Those most vulnerable to the effects of the lockdown were women aged over 45 years. The main concerns were the fear of being infected, the future of work, and isolation [11].

During the COVID-19 pandemic, economic changes and loneliness have increased psychological distress. Over a period of twelve months, spanning from April 2020 to March 2021, in Germany, in an online survey for members of the general population (n = 2703), 53.6% of respondents reported experiencing mild (34.2%) to severe psychological distress (19.4%). The increase in long-term mental health problems was associated with high federal government debt, high incidence of COVID-19 cases, low incomes, and the prevalence of loneliness. There was also an increased prevalence of psychological disorders in women and young people [12].

In healthcare workers (HPs), one of the groups most affected by the COVID-19 pandemic, mental health deteriorated with increased levels of stress, anxiety, and depression. This affected not only frontline hospital staff, but also out-of-hospital staff, albeit to a lesser extent. There was no difference between the first and second wave, and gender differences were found in both waves, with women most affected. Being in contact with COVID patients or those suspected of having COVID was strongly correlated with levels of stress and anxiety, while in the case of depressive symptoms, a strong correlation was found with having a clinical history of illnesses that could weaken defenses against infection. Stopping unpleasant emotions and thoughts was the coping strategy most frequently used by these HPs [13].

Finally, we present some thoughts about prevention. What have we learned about COVID-19 vaccination? A study carried out in Germany on 1037 elderly patients found that the intention or act of being vaccinated was strictly linked to perceptions of becoming infected, as well as perceptions of the severity of the potential long-term effects, the vaccine’s efficacy, and the benefits of vaccination. On the other hand, the negative side-effects were the main argument against getting the vaccine [14]. 

The fear of adverse events after COVID-19 vaccination must be alleviated through correct information derived from research, and many studies support this issue. The work carried out by Dziedzic and coll. Ref. [15] confirmed the safety of commonly administered vaccines against COVID-19, and only mild, self-resolving adverse events were observed. In this study involving healthcare workers, no major vaccine-related incidents were reported that could significantly affect everyday activities.
